# Polymeric mitral valve replacement in rheumatic heart disease: hemodynamic and early clinical outcomes from a dedicated sub-analysis of the India Surgical Trial

**DOI:** 10.3389/fcvm.2026.1870108

**Published:** 2026-08-17

**Authors:** J. Ryan Stanfield, Isaac George, Brian Whisenant, Anil Jain, Dasari Prasada Rao, Kaushal Pandey

**Affiliations:** 1 University of Utah, Salt Lake City, UT, United States; 2 Foldax, Inc., Salt Lake City, UT, United States; 3 New York Presbyterian Hospital-Columbia University Irving Medical Center, New York, NY, United States; 4 Intermountain Heart Institute, Salt Lake City, UT, United States; 5 EPIC Hospital, Ahmedabad, India; 6 Indo-US Superspeciality Hospital, Hyderabad, India; 7 PD Hinduja and Medical Research Centre, Mumbai, India

**Keywords:** clinical trial, global health, hemodynamic performance, India, mitral valve replacement, polymeric heart valve, rheumatic heart disease

## Abstract

**Background:**

Rheumatic heart disease (RHD) is a leading cause of cardiovascular morbidity in low- and middle-income countries (LMICs), disproportionately affecting young patients and women of childbearing age. Current valve options—mechanical and bioprosthetic—result in significant challenges in these populations, including the need for intensive anticoagulation monitoring or accelerated structural deterioration. This study evaluates a novel polymeric heart valve designed to overcome these limitations.

**Methods:**

The India Mitral Surgical Trial (CTRI/2023/02/049837) was a prospective, multicenter, single-arm trial involving 67 adult patients requiring mitral valve replacement. Patients were implanted with the polymer surgical mitral valve and followed for one-year with safety and hemodynamic assessments. Vitamin K antagonists were administered for a target INR of 2.5. This subgroup analysis focuses on patients with RHD.

**Results:**

The RHD subgroup, included 49 patients with a mean age of 39 years, of whom 33 (67%) were female. The trial achieved 100% technical success defined as successful implantation of the study valve with immediate satisfactory valve function and no intraoperative valve-related complications. At one-year, all-cause mortality for the RHD subgroup was 10.2% with no valve-related deaths and no incidence of structural valve deterioration or valve reintervention. The mitral inflow gradient decreased from 9.9 ± 5.1 mmHg at baseline to 4.2 ± 1.5 mmHg at discharge and 4.4 ± 1.7 mmHg at 1 year while the echo defined effective orifice area increased from 0.9 ± 0.5 cm^2^ at baseline to 1.6 ± 0.6 cm^2^ at discharge and remained stable at 1.5 ± 0.4 cm^2^ at 1 year. Thromboembolic events were comparable to commercial valves in the setting of a high percentage of patients requiring anticoagulation for preoperative atrial fibrillation.

**Conclusion:**

The polymeric valve demonstrated an acceptable safety profile and excellent hemodynamic stability at one-year. Further studies are needed to confirm durability and study anticoagulation reduction. Polymeric valves may be a promising option for patients with RHD.

## 1 Introduction

**Figure 1:**
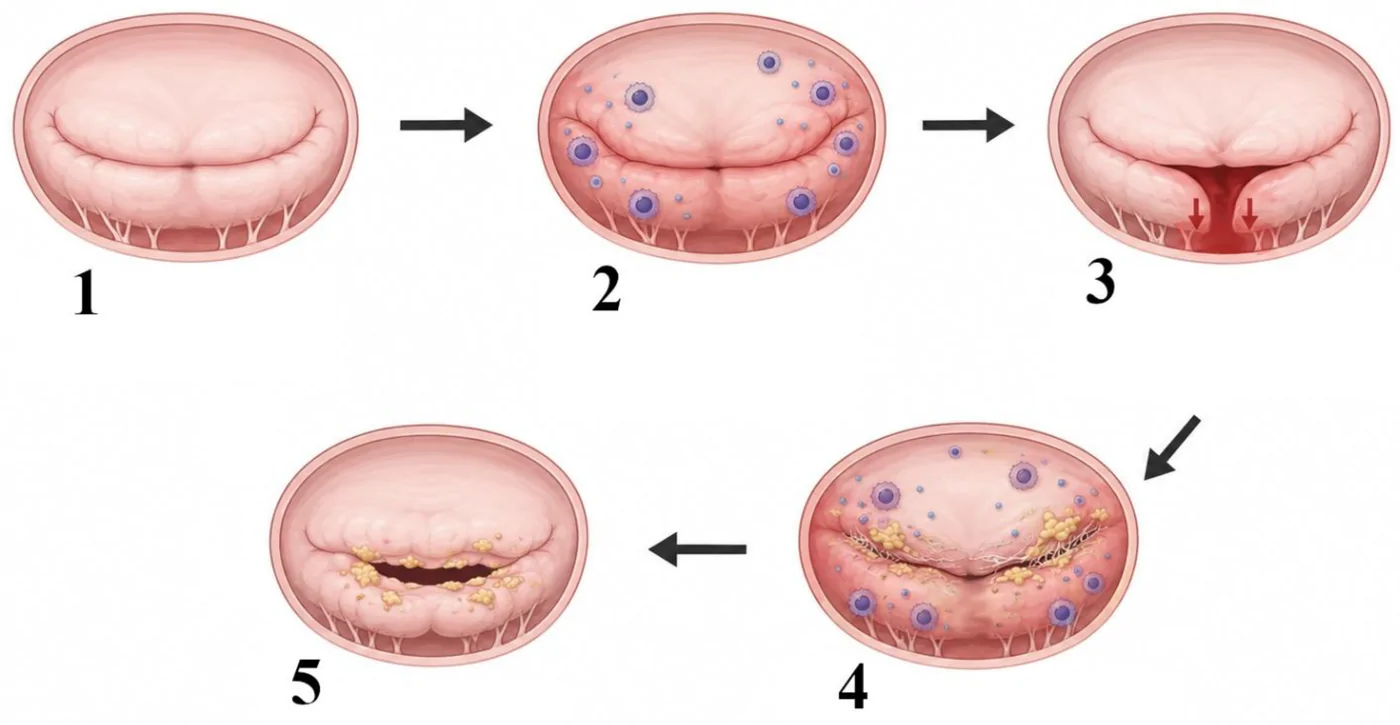
Accelerated valve calcification in patients with rheumatic heart disease. Beginning with a normal mitral valve (1), early rheumatic heart disease induces bioactivation and inflammation (2), leading initially to leaflet elongation and mitral regurgitation (3). Persistent inflammation promotes extracellular matrix remodeling, fibrotic leaflet thickening, and calcium deposition (4), progressively reducing leaflet flexibility. Ultimately, mitral stenosis develops (5) as leaflet thickening, commissural fusion, and calcification of the commissures and free edges restrict valve opening and impede blood flow.

Rheumatic heart disease (RHD) represents one of the most significant yet neglected cardiovascular diseases of our time, affecting over 24 million people globally and causing substantial morbidity and premature death, particularly among children and young adults in low- and middle-income countries (LMICs) (1–4). Rheumatic heart disease claims hundreds of thousands of lives annually, with India alone accounting for a disproportionate share of the global burden (4–6). The disease results from an aberrant autoimmune response to Streptococcus pyogenes infections, progressing through acute rheumatic fever to chronic valvular injury, most commonly affecting the mitral valve (7, 8). Unlike degenerative valve disease seen in older Western populations, RHD is a dynamic, inflammatory process that continues to affect the heart long after the initial infection (Figure 1).

For patients with severe RHD requiring valve replacement, the choice of prosthesis has historically posed challenging decisions. Mechanical valves offer durability but demand lifelong warfarin anticoagulation with frequent monitoring and at high levels, typically International Normalized Ratio (INR) target of 3 which is often impractical or impossible in resource-limited settings where most RHD patients reside (9–11). Conversely, bioprosthetic valves avoid the need for long-term anticoagulation but suffer from accelerated structural valve deterioration (SVD) in younger patients, frequently necessitating repeat operations within 10-15 years (9). For young RHD patients, particularly women of childbearing age who face additional risks from anticoagulation during pregnancy, neither option is ideal (12).

The management of RHD in LMICs is hampered by late diagnosis, poor adherence to secondary penicillin prophylaxis, and limited access to specialized cardiac surgery (3, 11). When surgery is available, the decision between mechanical and bioprosthetic valves is complicated by the patient's inability to access reliable anticoagulation monitoring or the high probability of early bioprosthetic failure (11). The lack of a “fit-and-forget” valve solution remains the primary bottleneck in improving RHD outcomes globally.

While mechanical valves are highly durable, their requirement for lifelong warfarin therapy is a significant barrier for RHD patients in rural or low-resource settings (9). Fluctuating INR levels due to poor access to medicine, dietary restrictions and variations, poor compliance, or lack of laboratory facilities lead to high rates of both thromboembolic events and major bleeding (13). In LMIC populations with RHD, prosthetic valve thrombosis occurs in approximately 10-12% of patients within a few years of surgery, substantially higher than the ∼0.1-5.0% per patient-year rates reported in well-controlled settings (14, 15). These events are frequently linked to subtherapeutic anticoagulation and carry high mortality (∼10-40%), often presenting as life-threatening “clotted valve” emergencies requiring urgent intervention (10).

Tissue valves are often selected to avoid anticoagulation, but their durability is significantly limited in young patients, particularly those with rheumatic heart disease. Structural valve deterioration—driven by accelerated calcification and leaflet degeneration—occurs earlier in younger populations, with median failure times of approximately 8–10 years and substantially reduced freedom from durability beyond a decade (16). In patients with rheumatic heart disease undergoing mitral valve replacement, bioprosthetic valves are associated with a ∼4–5-fold higher risk of reoperation compared with mechanical valves. In a large national cohort, 10-year reoperation rates were 8.9% for bioprosthetic valves versus 0.93% for mechanical valves, representing nearly a tenfold absolute difference (17). The metabolic and immunological environment of a young patient, especially one with active RHD, appears to trigger a more aggressive calcification response in bovine and porcine tissues (12). In addition to calcification, chronic inflammation and fibrosis in RHD may accelerate prosthetic valve dysfunction in younger patients. A summary comparing surgical valve replacement options for patients with rheumatic heart disease is shown in Table 1.

**Table 1:** Comparative valve options for RHD patients.

Valve type	Key advantages	Key limitations	RHD-specific considerations
Mechanical valves 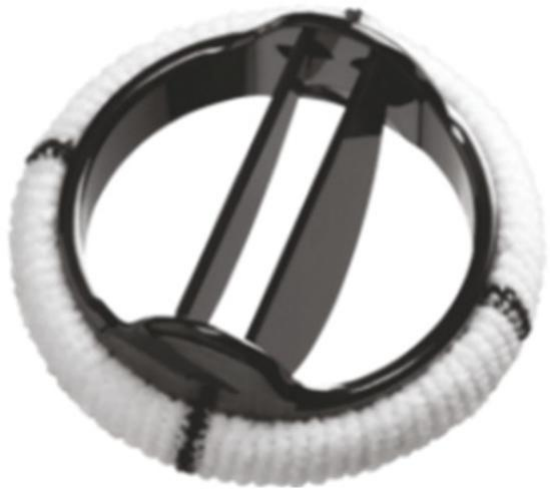	• Excellent durability (often >20–30 years) • Low structural failure rates • Established long-term clinical data	• Lifelong anticoagulation (warfarin) required • INR monitoring burden • High risk of thromboembolism if subtherapeutic • Risk of major bleeding (incl. intracranial hemorrhage) • Contraindicated/complex in pregnancy	• Major challenge in LMICs due to poor INR control (TTR ∼20–40%) • High rates of valve thrombosis and bleeding complications • Often problematic in young women (pregnancy risks)
Bioprosthetic (tissue) valves 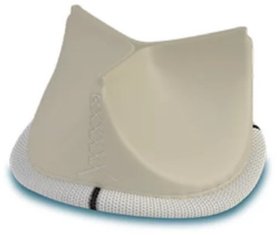 Valve image courtesy of Abbott Laboratories	• No lifelong anticoagulation (beyond early postop) • Lower bleeding risk • Safer for pregnancy • Easier management in low-resource settings	• Limited durability due to structural valve deterioration (SVD) • Accelerated calcification in younger patients • Reoperation typically required in ∼8–12 years (often sooner in RHD)	• Particularly poor durability in young RHD patients (mitral position) • ∼4–5× higher reoperation rates vs mechanical valves • Leads to multiple lifetime surgeries in pediatric/young adult populations
Polymeric valves (emerging) 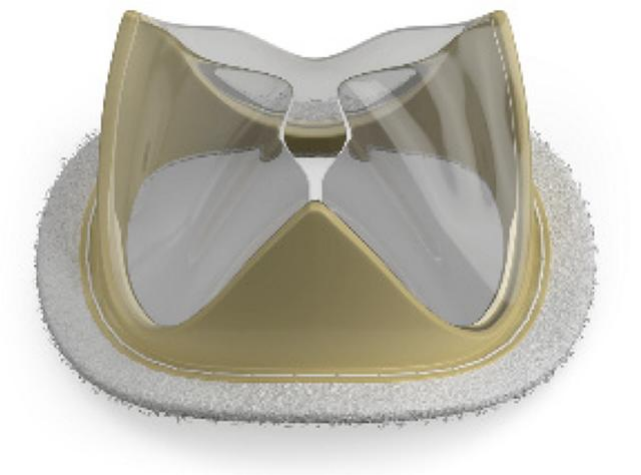 Valve image courtesy of Foldax, Inc.	• Potential for mechanical-like durability without anticoagulation • Resistance to calcification (design-dependent) • Tunable material properties • Potential for lower thrombogenicity	• Limited long-term human data • Durability not yet fully proven clinically • Regulatory pathway still evolving • Unknown long-term failure modes (fatigue, creep, etc.)	• Highly attractive for RHD due to: – Avoidance of INR burden – Potential durability in young patients • Still largely investigational/early clinical adoption
Tissue-engineered valves (TEHV) 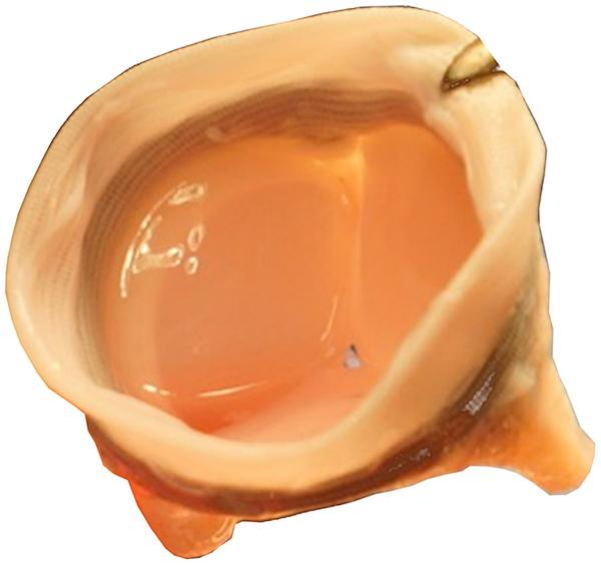	• Potential for growth and remodeling (ideal for pediatric patients) • May eliminate need for anticoagulation • Conceptually “living valve” with repair capability	• Early-stage technology • Limited clinical translation to date • Manufacturing complexity • Risk of immune response or scaffold failure • Long-term durability unknown	• Could be transformative for young RHD patients (especially children) • Addresses need to avoid repeat surgeries due to growth • Currently not widely available clinically

Mechanical valves adapted from “Bileaflet mechanical heart valve (BMHV): St. Jude Masters HP series valve” by Woojin Kim, Haecheon Choi, Jihoon Kweon, Dong Hyun Yang and Young-Hak Kim, licensed under CC BY 4.0. Tissue-engineered valves (TEHV) adapted from “Figure 6. (A) An atrial view of the H-TEHV before surgical implantation into the sheep's mitral position” by Sutherland et al., licensed under CC BY 4.0.

RHD disproportionately affects women of childbearing age. For these patients, mechanical valves present a double-edged sword: warfarin is teratogenic during the first trimester, yet switching to heparin is associated with a high risk of valve thrombosis. Conversely, bioprosthetic valves fail even faster during pregnancy due to hemodynamic and hormonal changes, often requiring urgent surgery during or shortly after gestation (18). There is a critical unmet need for a valve that provides the durability of a mechanical prosthesis without the teratogenic and hemorrhagic risks of high-intensity anticoagulation.

The TRIA heart valve (Foldax; Salt Lake City, USA) represents a radical departure from traditional bioprosthetic and mechanical designs by utilizing a uniquely formulated, biostable siloxane-based polyurethane-urea (SiPUU, LifePolymer™) (19). Unlike bovine or porcine tissue, which requires glutaraldehyde fixation, a process that introduces residual toxicity and promotes intrinsic calcification, the SiPUU polymer is synthesized to be inherently biocompatible and biostable (20). Preclinical toxicologic assessments have confirmed that the SiPUU polymer is non-mutagenic, non-hemolytic, and produces no significant complement activation. Furthermore, studies in ovine models demonstrated no evidence of leaflet calcification and only minimal fibrinous deposition, contrasting sharply with the early mineral deposition often seen in bioprosthetic tissues. The material's biostability has been further verified in non-human primate arteriovenous shunt models, showing excellent resistance to biodegradation and thrombus formation (21).

Given these limitations of existing valve prostheses, there remains a critical unmet need for durable, scalable solutions tailored to the rheumatic heart disease population. Polymeric valve technology represents a novel approach toward addressing this need. The India Mitral Surgical Trial provides the first multicenter clinical evaluation of a polymeric mitral valve in a predominantly rheumatic population (22). In this study, we report one-year clinical and hemodynamic outcomes in the rheumatic heart disease subgroup to assess early safety and performance in this high-risk population.

## 2 The India mitral surgical trial: methods

The India Mitral Surgical Trial (CTRI/2023/02/049837 also cross-listed as NCT06191718) was a prospective, multicenter, single-arm clinical trial conducted at eight specialized cardiac centers in India. From April to November 2023, adult patients requiring surgical mitral valve replacement (SMVR) were screened by an independent physician committee. While the parent trial enrolled 67 patients with mixed mitral valve etiologies, the present manuscript focuses specifically on the subgroup with rheumatic heart disease (*n* = 49, 73% of the total cohort). The present analysis represents a defined etiologic subgroup of patients with rheumatic heart disease; patients with non-rheumatic mitral valve disease enrolled in the parent trial were not included in this analysis. The study was designed to evaluate both safety events (per VARC-3 criteria) and valve performance (via echocardiographic and CT imaging) (22). Adverse events were adjudicated by an independent clinical events committee. Written informed consent was obtained from each study patient, and institutional review board approval was granted at each site.

The surgical procedure involved standard median sternotomy and cardiopulmonary bypass with interrupted sutures for all patients. Patients were maintained on a vitamin K antagonist (warfarin) with a target INR of 2.5 for the first year (22).

Statistical analyses were performed for the rheumatic heart disease (RHD) subgroup (*n* = 49) using methods consistent with the parent trial (22). Early outcomes are presented as incidence rates. Competing risk methods, such as cumulative incidence functions, were considered; however, Kaplan–Meier estimates were used to maintain consistency with the primary trial analysis, from which event-free probabilities at one-year were derived. Greenwood's formula was used to calculate 95% confidence intervals on Kaplan–Meier event-free probabilities. As a sensitivity analysis, cumulative incidence functions were calculated for ischemic stroke and valve thrombosis using all-cause mortality as a competing risk. The resulting estimates were compared with Kaplan–Meier event rates to assess the potential impact of competing events on interpretation.

Echocardiographic data were analyzed by an independent core laboratory and summarized as mean ± standard deviation for continuous variables. Hemodynamic parameters were evaluated at baseline, discharge, and scheduled follow-up intervals (30 days, 90 days, 180 days, and 1 year). Analyses of hemodynamic changes were performed using paired data from patients with available measurements at both baseline and the corresponding follow-up time point. The number of patients contributing echocardiographic data varied across time points due to incomplete follow-up or missing data; no imputation was performed, and analyses were conducted using available data only. Changes in hemodynamic parameters from baseline to follow-up were assessed using paired Student's t-tests. To account for multiple comparisons across time points, Bonferroni correction was applied as needed. Although linear mixed-effects modeling represents an alternative approach for longitudinal data analysis, paired comparisons were used in this study to maintain consistency with the statistical methodology of the primary trial analysis. A sensitivity analysis using a linear mixed-effects model yielded comparable results and did not change the interpretation. Hemodynamic data were additionally stratified by valve size for descriptive analysis. Functional status (New York Heart Association class) is reported as the proportion of patients in each class at baseline and at one-year. A *P* value <0.05 was considered statistically significant.

## 3 Results: one-year clinical and hemodynamic outcomes

Baseline characteristics for patients with rheumatic heart disease are detailed in Table 2. The RHD subgroup was younger than the full trial cohort, with a mean age of 39 years (range 19–58), and women comprised 33 (67%) of the 49 participants affected by RHD, with 24 women being less than age 45, generally considered of childbearing age. The mean Body Mass Index (BMI) was 22.6 kg/m^2^, and the mean Body Surface Area (BSA) was 1.5 m^2^. The mean Society of Thoracic Surgeons (STS) Predicted Risk of Mortality score was 1.3%, and baseline assessments showed that 47% of patients were in NYHA functional class III or IV. Preoperative assessment showed that 27% of patients suffered from pure stenosis, 30% from regurgitation, and 43% from mixed valvular disease (22).

**Table 2:** Baseline patient characteristics.

Demographics (*N* = 49)	
Age, y	39.0 ± 10.5
	37 (19-58)
Age range, y	
≤25	6 (12.2)
26-35	15 (30.6)
36-45	13 (26.5)
46-55	10 (20.4)
>55	5 (10.2)
Height, cm	156.3 ± 10.0
Weight, kg	55.2 ± 13.6
BMI, kg/m2	22.6 ± 5.0
BSA, m2	1.5 ± 0.2
Sex	
Female	33 (67.3)
Male	16 (32.7)
Women of childbearing age	20 (40.8)
STS Risk Score, %	1.3 ± 0.7
Functional assessments and laboratory values	
NYHA functional class III or IV	23 (46.9)

Values are mean ± SD, median (range), or n (%).

The trial achieved 100% technical success defined as successful implantation of the valve with immediate satisfactory valve function and no intraoperative valve-related complications. Selected events and outcomes are detailed in Table 3. Given the small number of observed events, corresponding confidence intervals are wide. Five deaths occurred during the one-year study period (10.2% of RHD subgroup population, but result in a 10.4% probability of occurrence using Kaplan–Meier estimates). Causes included one intraoperative left ventricular free wall rupture and two deaths from sepsis. Two additional deaths occurred outside the hospital and were classified by the independent Clinical Events Committee as deaths of undetermined cause due to limited clinical information and absence of autopsy data. In both cases, no prior evidence of valve dysfunction had been documented on follow-up assessment. That said, the relationship of these two events to the study device or procedure could not be definitively established.

**Table 3:** Selected events and outcomes.

Event	Early (≤30 d)	Cumulative at 1 yr^a^, (%)	Probability event-free at 1 yr^a^, % [95% CI]
All-cause mortality	1 (2.1)	5 (10.4)	89.6% [80.8-98.3%]
Valve or cardiovascular related mortality	0 (0)	0 (0)	100%
Valve reintervention/reoperation	0 (0)	0 (0)	100%
Study valve explant	0 (0)	0 (0)	100%
Structural valve deterioration	0 (0)	0 (0)	100%
Nonstructural valve dysfunction	0 (0)	1 (2.1)	97.9%
Thromboembolism	1 (2.1)	4 (8.8)	91.2% [83.0-99.5%]
Ischemic stroke	1 (2.1)	2 (4.4)	95.6% [89.3-100%]
Hemorrhagic stroke	0 (0)	0 (0)	100%
Transient ischemic attack	0 (0)	0 (0)	100%
Valve thrombosis	0 (0)	2 (4.5)	95.5% [89.2-100%]

a These percentages are Kaplan–Meier estimates at the specific timepoint and thus do not equal the number of patients with events divided by the total number of patients. 95% confidence intervals (CI) is also included for probabilities of event-free and one-year.

Values are n (%) unless otherwise indicated.

During follow-up, thromboembolic and bleeding events were infrequent and occurred in the context of anticoagulation variability. Two patients experienced ischemic cerebrovascular events (corresponding to a 1-year Kaplan–Meier incidence of 4.4%), and two cases of valve thrombosis were observed (4.5%). One additional patient experienced a major bleeding event (2.1%) [LV rupture]. Both ischemic strokes occurred during periods of subtherapeutic anticoagulation (INR of 1.3 and 1.7). One patient underwent thrombolytic therapy for an acute cortical infarction, while the other patient was found to have subacute ischemic changes on brain magnetic resonance imaging and was managed conservatively with adjustment of vitamin K antagonist therapy. Neurologic symptoms resolved in all affected patients without residual deficits. Sensitivity analyses using cumulative incidence functions with all-cause mortality treated as a competing risk yielded estimated one-year incidences of 4.1% for ischemic stroke and 4.1% for valve thrombosis, which were similar to the corresponding Kaplan–Meier estimates of 4.4% and 4.5%, respectively, and did not alter interpretation of the findings.

Both episodes of valve thrombosis were detected incidentally at routine follow-up visits, with one at 90 days and one at one-year, in asymptomatic patients with documented subtherapeutic anticoagulation (INR values of 1.0 and 1.5). Prompt hospitalization and thrombolytic treatment resulted in complete thrombus resolution with restoration of normal valve hemodynamics in both cases. One patient was readmitted at 30 days for heart failure symptoms; transthoracic echocardiography demonstrated a normally functioning prosthetic valve. The primary safety endpoint of structural valve deterioration (SVD) was 0%, and no patients required valve reintervention during the first year of follow-up.

**Figure 2:**
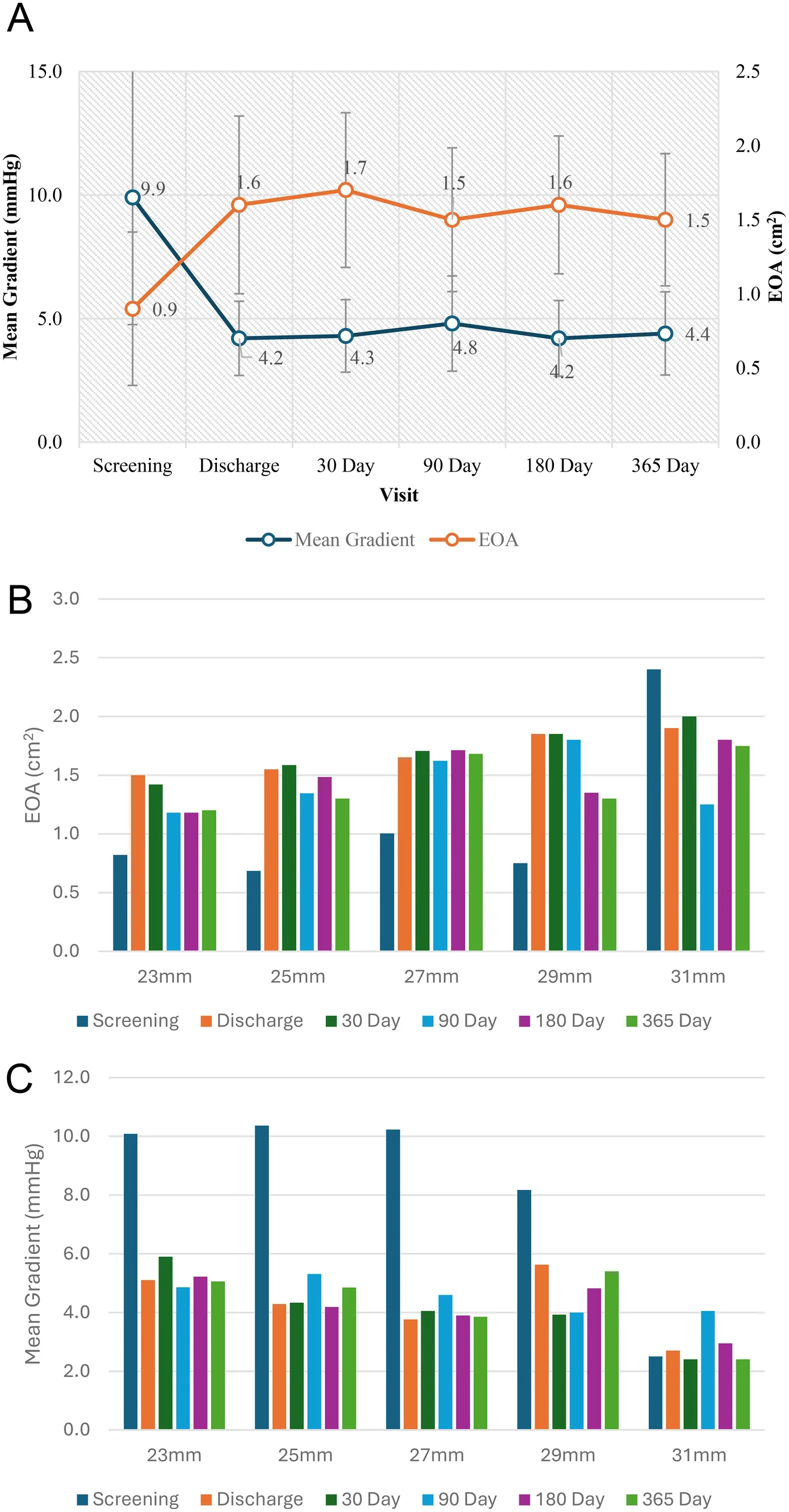
Echocardiography-derived hemodynamics. **(A)** In RHD patients with paired echocardiograms, a significant increase in the effective orifice area (EOA) with corresponding significant decrease in mean mitral gradient was registered across the study period (Average values shown with standard deviation error bars). As expected, larger valve sizes were associated with **(B)** higher EOA and **(C)** lower mitral gradient across the entire study period. Average values are reported for each time point and valve size. Valve size-stratified analyses are descriptive only and are not intended for formal comparison due to small sample sizes within each group.

The TRIA valve demonstrated excellent hemodynamic stability. The mean EOA increased from 0.9 ± 0.5 cm^2^ pre-implant to 1.6 ± 0.6 cm^2^ at discharge and remained stable at 1.5 ± 0.4 cm^2^ at 1 year, while mean pressure gradient decreased from 9.9 ± 5.1 mmHg pre-implant to 4.2 ± 1.5 mmHg at discharge and 4.4 ± 1.7 mmHg at 1 year (Figure 2).

## 4 Discussion and future directions

### 4.1 Addressing the unique needs of RHD patients

The absence of SVD at one-year in a young RHD population is highly significant. In younger patients, bioprosthetic valves typically show signs of early calcification within 24 months. The stability of the polymer material, as confirmed by early human case reports showing no leaflet thrombus or calcification at 6 months and stable gradients at 12 months (22), provides an early signal of favorable short-term performance and potential to reduce the need for repeat interventions, although long-term durability remains to be established with extended follow-up.

The magnitude and duration of hemodynamic improvement observed in the rheumatic subgroup are clinically significant. Patients with rheumatic heart disease entered the trial with severe baseline obstruction, as reflected by a mean EOA of 0.9 cm^2^ and mean pressure gradient of 9.9 mmHg. Following mitral valve replacement, EOA doubled at discharge and remained stable through one-year, while transmitral pressure gradients fell by 58% and did not increase over time. This stability is particularly noteworthy in rheumatic disease, where early pannus formation, fibrosis, and accelerated calcification frequently lead to progressive gradient increase within the first year after implantation of tissue valves. The consistency of hemodynamic trends between the rheumatic subgroup and the full trial cohort supports the generalizability of these findings within the context of early follow-up. Although hemodynamic data are presented by valve size for descriptive purposes, the small number of patients within each size category and lack of adjustment for baseline characteristics limit the ability to draw comparative conclusions.

The one-year findings from the India Mitral Surgical Trial gain additional context when viewed alongside emerging long-term human polymer valve data. Early clinical experience with the same LifePolymer leaflet material has demonstrated sustained hemodynamic performance and intact leaflet structure on serial echocardiography and computed tomography, with no evidence of calcification, pannus related leaflet restriction, or material breakdown (22, 23). These observations are particularly salient for rheumatic heart disease, in which accelerated failure of tissue valves is driven not only by calcification but also by chronic inflammation and fibrotic overgrowth. While extended follow-up of mitral implants remains essential, the available human durability data supports the hypothesis that this polymeric leaflet technology may be more resilient to the hostile biological milieu characteristic of rheumatic heart disease.

The demographic profile of the rheumatic cohort highlights the clinical relevance of these findings. With nearly two-thirds of patients being female and a mean age of 39 years, this population represents individuals likely to face multiple decades of valve related management, including potential pregnancy. In this context, the sustained hemodynamic performance observed at one-year without evidence of early deterioration addresses a critical gap left by existing mechanical and bioprosthetic options, which often impose unacceptable reproductive or reintervention risks for young women with rheumatic heart disease. Although a substantial proportion of this cohort was of childbearing age, pregnancy outcomes were not assessed within the one-year study period, and the impact of polymeric valve implantation during pregnancy remains an important area for future investigation.

Thromboembolic events and valve thrombosis were infrequent in this cohort but require careful interpretation. In the rheumatic subgroup, four events (two ischemic strokes and two cases of valve thrombosis) were observed and occurred in the setting of subtherapeutic anticoagulation, with INR values ranging from 1.0 to 1.7 relative to a target of 2.5. A similar pattern was observed in the full trial cohort, in which five thromboembolic events occurred among 67 patients (22), supporting the consistency of these findings across populations.

While these observations are consistent with inadequate anticoagulation, causality cannot be definitively established. Thromboembolic risk in patients with rheumatic heart disease is multifactorial and may be influenced by medication adherence, dietary variability, comorbid conditions, atrial fibrillation burden, and challenges in INR monitoring, particularly in resource-limited settings. Importantly, all patients in this study were managed with standard vitamin K antagonist therapy, and the present data do not allow conclusions regarding alternative anticoagulation strategies or the intrinsic thrombogenic profile of the valve material.

While this trial utilized standard anticoagulation, the excellent hemocompatibility observed in preclinical primate shunt models and the human clinical data suggest that further investigation into reduced anticoagulation strategies is warranted in future prospective studies (21, 22). For the women in this subgroup who are of childbearing age, the transition to a low-intensity or even aspirin-only protocol could represent an important advancement in RHD management if demonstrated to be safe in future studies by reducing teratogenic risks and catastrophic bleeding (12, 18).

Appropriate valve sizing is particularly relevant in rheumatic heart disease, where annular distortion, fibrosis, and commissural fusion are common and may complicate intraoperative assessment. In contrast to traditional bioprosthetic paradigms that often favor upsizing to compensate for anticipated effective orifice area reduction, polymeric valve performance depends on true sizing to the native annulus to preserve intended leaflet kinematics and flow characteristics. Computational modeling and early clinical experience indicate that maintaining the designed geometric relationship between the valve frame and annulus supports optimal strain distribution across polymer leaflets and avoids unnecessary mechanical stress (22–24). In rheumatic mitral surgery, where annuli are frequently small and rigid, a true-sizing strategy may simplify surgical decision-making while preserving favorable early hemodynamic performance.

### 4.2 Advantages over conventional prostheses

The 1-year results from India suggest that polymeric valves may match or exceed the hemodynamic performance of the best available bioprostheses while offering a material profile that may help mitigate mechanisms of SVD seen in young patients. Unlike bioprosthetic valves that show early gradient increases, the TRIA valve maintained stable gradients through 12 months (22). Robotic manufacturing ensures a level of quality control that exceeds the variability inherent in animal-tissue harvesting and hand-sewing, potentially reducing the incidence of non-structural valve dysfunction.

In the context of rheumatic heart disease, stability of post implant gradients is arguably a more meaningful endpoint than absolute hemodynamic comparison to degenerative cohorts. Progressive obstruction following bioprosthetic mitral valve replacement has been well described in young rheumatic patients, often within the first several years after surgery (17). The absence of gradient creep at one-year in this rheumatic cohort suggests that polymeric leaflet material may mitigate early inflammatory or fibrotic mechanisms that contribute to rapid functional deterioration of tissue valves in this population.

The TRIA valve is supplied in a “dry state,” requiring no glutaraldehyde rinsing or complex preparation before implantation (22, 23). The dry-state storage and robotic scalability address two major barriers to cardiac surgery in LMICs: logistics and cost. The ability to manufacture these valves in high-volume, automated facilities could eventually lower the unit cost, making advanced heart valve therapy accessible to the millions of RHD patients currently left untreated. The absence of glutaraldehyde eliminates the need for complex intraoperative rinsing, reducing the time on cardiopulmonary bypass – a critical factor in improving outcomes in sick RHD patients.

Clinical data from the US-FDA Early Feasibility Study and subsequent computational modeling have established a critical departure from traditional surgical practice: there is no need to oversize the TRIA valve. Standard bioprosthetic practice often involves oversizing to compensate for eventual reduction in effective orifice area (EOA) due to pannus or calcification. However, the TRIA valve relies on a low elastic modulus and specific flow-field optimizations that are disrupted by oversizing. Computational fluid dynamics show that a precise fit is mandatory to maintain the intended leaflet kinematics and avoid imperfect hemodynamic outcomes. Oversizing can lead to increased mechanical strain on the leaflets and potentially compromise the long-term biostability of the polymer (21, 24). Extensive *in vitro*, *in vivo*, and early human clinical evidence demonstrates that the SiPUU polymer leaflets do not calcify (19, 20, 22, 23), eliminating one of the primary motivations for oversizing in traditional bioprosthetic valves.

Rheumatic mitral valve disease disproportionately affects young women, often during their reproductive years, amplifying the clinical consequences of valve choice through pregnancy related anticoagulation risk and early prosthetic failure. In the present clinical experience, the rheumatic subgroup was predominantly female (67%) with a mean age of 39 years, underscoring the urgent need for valve technologies designed specifically for young patients who must balance durability, hemodynamics, and reproductive health considerations.

The clinical implications of these findings must be considered within the broader context of healthcare access in low- and middle-income countries. Rheumatic heart disease remains highly prevalent in India, with a substantial population progressing to severe valvular disease requiring intervention. Although surgical valve replacement is an established treatment, the number of procedures performed annually is markedly lower than the estimated disease burden, reflecting persistent limitations in access to specialized cardiac care. Published estimates suggest that over 30,000 surgical heart valve procedures are performed annually in India, whereas the population of patients with advanced rheumatic valvular disease is substantially larger (25). In addition, although the cost of valve surgery is lower than in high-income settings, out-of-pocket expenses remain significant for many patients, and access to publicly funded care is variable. Accordingly, the real-world impact of emerging valve technologies will depend not only on clinical performance but also on health system factors including affordability, infrastructure, and access to longitudinal follow-up. These considerations warrant further study.

### 4.3 Future research

The promising results from the India Mitral Surgical Trial provide a foundation for several critical research priorities aimed at optimizing RHD care:

1. Extended Follow-up of Mitral Cohort: We aim to follow as many of the original 67 surgical mitral trial patients as possible to assess long-term durability. This longitudinal study is contingent upon re-consenting participants, and we anticipate that the final follow-up cohort may be smaller than the initial enrollment. Long-term biostability and resistance to pannus formation will be key metrics.
2. Post-Commercial Registry: Following commercial approval, we plan to establish a comprehensive registry in India to track approximately 100 new patients. This will provide real-world data on valve performance and safety across a broader clinical spectrum, including patients with concomitant procedures and multiple valve disease.
3. Five-Year Aortic Outcomes: Continued monitoring of the USFDA EFS patients implanted with surgical aortic valves is ongoing, with a focus on those reaching the five-year milestone to evaluate mid-term biostability and hemodynamic stability (23). This will provide the first long-term human data on the SiPUU polymer material.
4. Reduced Anticoagulation Trial: A dedicated trial may be proposed to evaluate a reduced anticoagulation protocol, targeting an INR range of 1.5–2.0 (or even 0-2.0 in selected cases) in eligible patients. This is perhaps the most critical research direction for the LMIC population, where warfarin-related morbidity is a major cause of post-surgical mortality (26).
5. Advanced Calcification Imaging: To definitively assess the calcification resistance of the polymer leaflets, we propose a specialized study using novel biotracers combined with PET/CT scanning to detect early-stage mineral deposition at the molecular level. This would provide objective evidence of the biostability of the SiPUU polymer compared to animal tissue.

This analysis has several important limitations. The study was derived from a prospective, single-arm clinical trial without a randomized comparator group, limiting direct comparison with mechanical or bioprosthetic valves. The present report is further restricted to a subgroup of patients with rheumatic heart disease, resulting in a relatively small sample size that may limit generalizability. Follow-up is currently limited to one-year, precluding assessment of long-term durability, structural valve deterioration, and late clinical outcomes. In addition, no definitive conclusions can be drawn regarding the safety or feasibility of reduced anticoagulation strategies. Longitudinal hemodynamic analyses were based on available follow-up data and may be subject to bias due to random missingness. Two deaths in this cohort were classified as of undetermined cause due to limited available clinical information and lack of autopsy data. As such, a cardiovascular or valve-related etiology cannot be definitively excluded, representing an inherent limitation of follow-up in real-world settings. Larger studies with longer follow-up and comparative designs will be required to fully define the long-term performance and optimal management strategies for polymeric mitral valves in rheumatic populations.

## 5 Conclusions

In this dedicated analysis of patients with rheumatic heart disease undergoing mitral valve replacement, the TRIA polymeric mitral valve demonstrated marked and sustained hemodynamic improvement from a severely obstructed baseline, with stable effective orifice area and low transmitral pressure gradients through one-year. These findings suggest that polymeric valve technology may be well suited to the unique biological and hemodynamic challenges of rheumatic heart disease, offering sustained relief of obstruction without early degeneration in a young patient population. Further follow-up will be essential to confirm long-term durability and to define optimal anticoagulation strategies, particularly for patients in resource-limited settings.

## Data Availability

Requests to access the raw data supporting the conclusions of this article should be directed to ryan.stanfield@utah.edu.
